# Supplementary material for: Individual differences in learning and decision-making: the role of COMT Val158Met polymorphism in transitive inference
Source: Exp Brain Res. 2026 Feb 26;244(4):52. doi: 10.1007/s00221-026-07247-4 (PMC12946361; doi:10.1007/s00221-026-07247-4)
Supplement: Supplementary file 1 — Supplementary Material 1 [file 221_2026_7247_MOESM1_ESM.docx]

**SUPPLEMENTARY MATERIALS**

**Supplementary Figure S1**
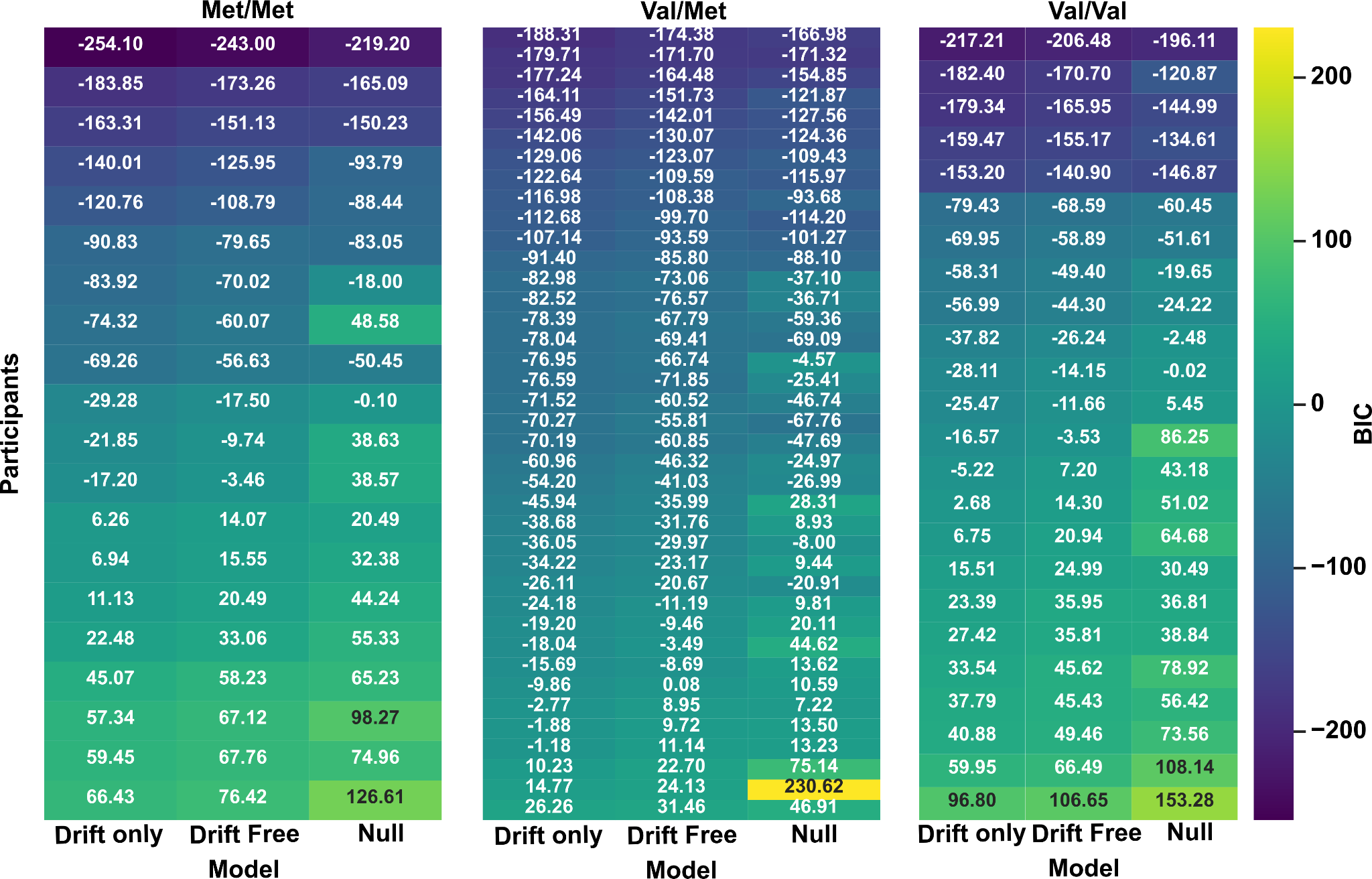


**Supplementary Figure S1**. BIC Values of Test Phase for each participant: The Figure lists, for every genotype and participant, the BIC obtained when fitting three alternative DDMs to the RT distributions recorded in the test phase: (i) a Null model in which no decision parameter varies with SD, (ii) a Drift-only model allowing the drift-rate to scale linearly with SD, and (iii) a Drift-free model allowing the drift-rate to change with SD without a linear constraint. Cells are colour-coded with a blue-yellow gradient in which the darkest color marks the lowest (best) BIC within each row

**Supplementary Figure S2**
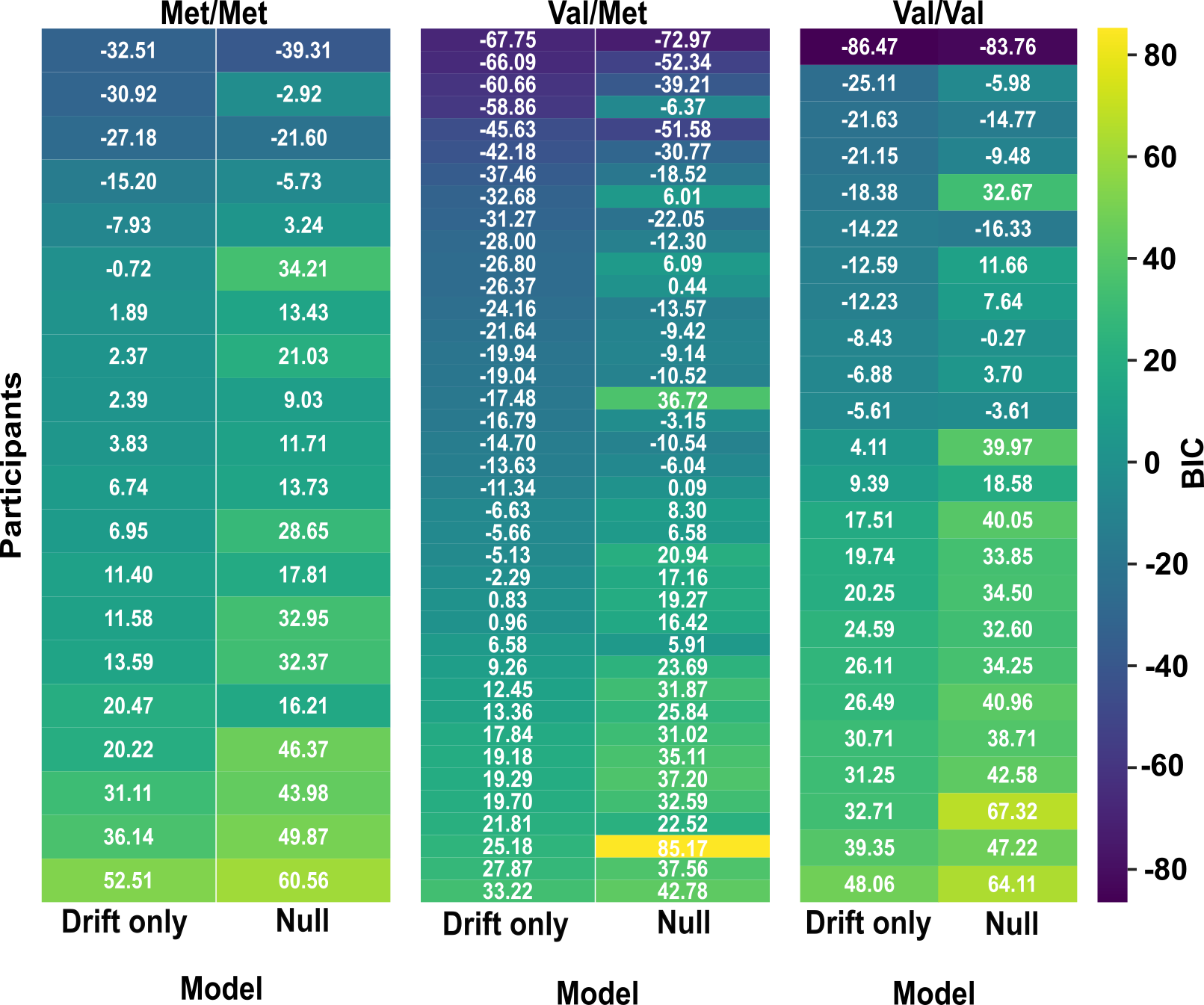


**Supplementary Figure S2**. BIC Values of the Last Learning Block for each participant. The Figure lists, for every genotype and participant, the BIC obtained when fitting two alternative DDMs to the RT distributions recorded in the final learning block: (i) a Null model in which no decision parameter varies with the pair and (ii) a Drift-only model allowing the drift to change with the pair, reflecting a U shape. Cells are colour-coded with a blue-yellow gradient in which the darkest color marks the lowest (best) BIC within each row

**Supplementary Figure S3**
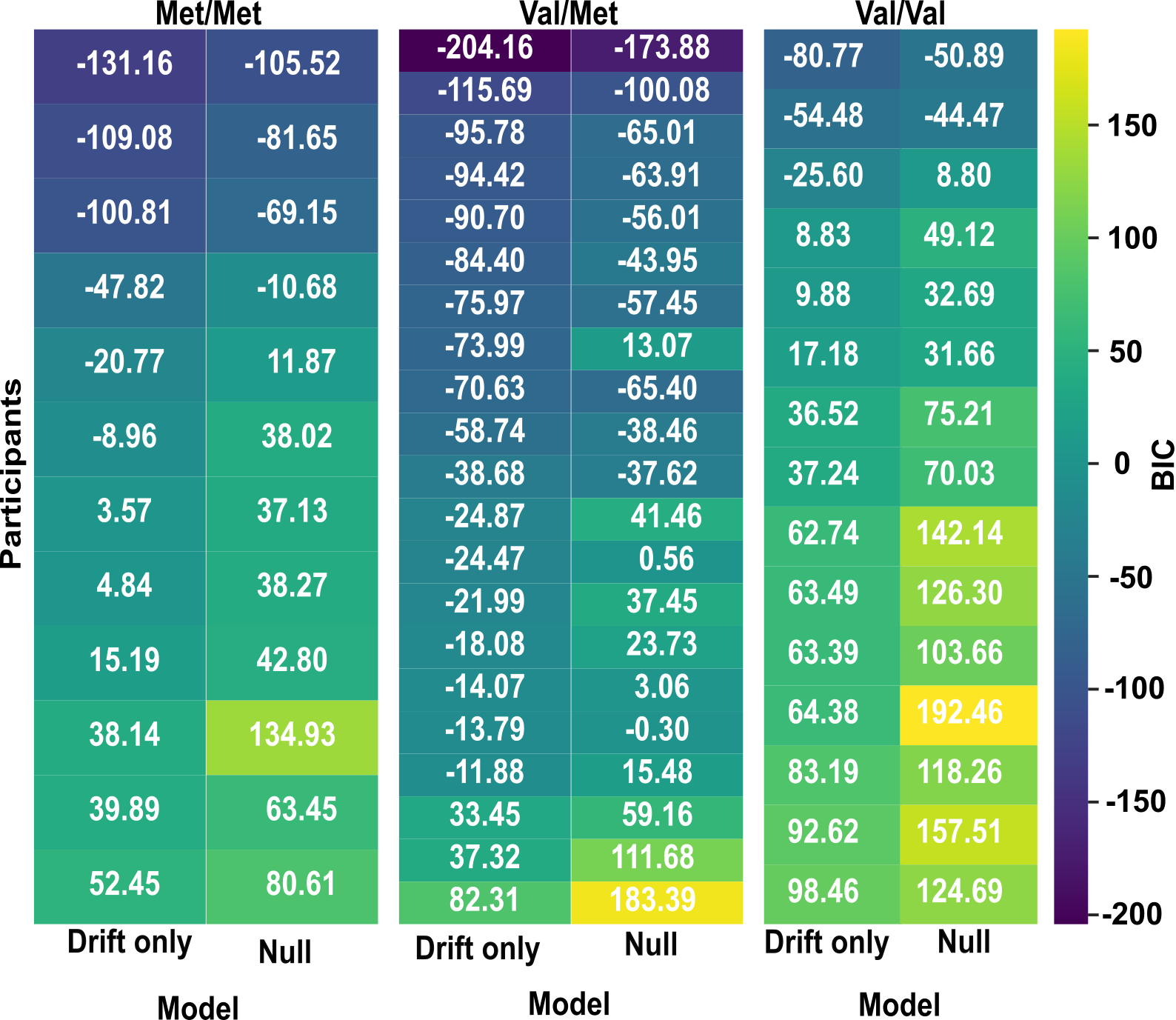


**Supplementary Figure S3**.BIC Values of Reverse Phase for each participant. The Figure lists, for every genotype and participant, the BIC obtained when fitting two alternative DDMs to the RT distributions recorded in the reversed phase: (i) a Null model in which no decision parameter varies with SD and (ii) a Drift-only model allowing the drift-rate to scale linearly with SD. Cells are colour-coded with a blue-yellow gradient in which the darkest color marks the lowest (best) BIC within each row
